# Selection Signatures Analysis Reveals Genes Associated with High-Altitude Adaptation in Tibetan Goats from Nagqu, Tibet

**DOI:** 10.3390/ani10091599

**Published:** 2020-09-08

**Authors:** Meilin Jin, Jian Lu, Xiaojuan Fei, Zengkui Lu, Kai Quan, Yongbin Liu, Mingxing Chu, Ran Di, Caihong Wei, Huihua Wang

**Affiliations:** 1 Institute of Animal Science, Chinese Academy of Agricultural Sciences, Beijing 100193, China; jmlingg@163.com (M.J.); 18409481571@163.com (X.F.); mxchu@263.net (M.C.); dirangirl@163.com (R.D.); 2 National Animal Husbandry Service, Beijing 100193, China; lujian34@163.com; 3 Lanzhou Institute of Husbandry and Pharmaceutical Sciences, Chinese Academy of Agricultural Sciences, Lanzhou 730050, China; luzengkui@caas.cn; 4 College of Animal Science and Technology, Henan University of Animal Husbandry and Economy, Zhengzhou 450046, China; quankai1115@163.com; 5 Inner Mongolia Academy of Animal Husbandry Science, Hohhot 010031, China; ybliu117@126.com

**Keywords:** high-altitude adaptation, Tibetan goat, selection signal

## Abstract

**Simple Summary:**

In the process of domestication, goats have undergone long-term artificial and natural selection, leading to differences among goat breeds and leaving different selection traces on the genome. However, the genetic components underlying high-altitude adaptation remain largely unknown. Here, we genotyped four goat breeds using the Illumina Caprine 50K single nucleotide polymorphism (SNP) Chip. One highland breed (Tibetan goat) compared with three lowland breeds (Huanghuai goat, Taihang goat and Xinjiang goat) to identify the molecular basis of high-altitude adaptation. So, we investigated selection signatures using the *di* statistic of four goat breeds and some genes in Tibetan goats related to high-altitude adaptation were identified. In addition, q-PCR validated the gene expression level in Tibetan goats and Huanghuai goats. This information may be valuable for the study of the genetic uniqueness of Tibetan goats and increased understanding of the hypoxic adaptation mechanism of Tibetan goats on the plateau.

**Abstract:**

Tibetan goat is an ancient breed, which inhabits the adverse conditions of the plateaus in China. To investigate the role of selection in shaping its genomes, we genotyped Tibetan goats (Nagqu Prefecture, above 4500 m) and three lowland populations (Xinjiang goats, Taihang goats and Huanghuai goats). The result of PCA, neighbor-joining (N-J) tree and model-based clustering showed that the genetic structure between the Tibetan goat and the three lowland populations has significant difference. As demonstrated by the *di* statistic, we found that some genes were related to the high-altitude adaptation of Tibetan goats. Functional analysis revealed that these genes were enriched in the VEGF (vascular endothelial growth factor) signaling pathway and melanoma, suggesting that nine genes (*FGF2*, *EGFR*, *AKT1*, *PTEN*, *MITF*, *ENPEP*, *SIRT6*, *KDR*, and *CDC42*) might have important roles in the high-altitude adaptation of Nagqu Tibetan goats. We also found that the *LEPR* gene was under the strongest selection (*di* value = 16.70), and it could induce upregulation of the hypoxic ventilatory response. In addition, five genes (*LEPR*, *LDB1*, *EGFR*, *NOX4* and *FGF2*) with high *di* values were analyzed using q-PCR. Among them, we found that *LEPR*, *LDB1* and *FGF2* exhibited higher expression in the lungs of the Tibetan goats; *LEPR*, *EGFR* and *LDB1* exhibited higher expression in the hearts of the Huanghuai goat. Our results suggest that *LEPR*, *LDB1*, *EGFR* and *FGF2* genes may be related to the high-altitude adaptation of the goats. These findings improve our understanding of the selection of the high-altitude adaptability of the Nagqu Tibetan goats and provide new theoretical knowledge for the conservation and utilization of germplasm resources.

## 1. Introduction

In China, there are approximately 58 native domestic goat breeds that are distributed widely across a range of environments [1]. Tibetan goat is valued for its important genetic resources and has adapted to living on the Tibetan plateau (above 4000–5500 m). It is characterized by low partial oxygen, temperature and pressure, and high levels of ultraviolet radiation [2]. Through long-term natural selection, they have acquired stable genetic characteristics in physiology, biochemistry and morphology to adapt to the low-oxygen environment on the plateau [3]. Their genetic background fully demonstrates the unique adaptability of plateau species in the long process of evolution. In turn, this selection might have left genetic footprints in the Tibetan goat genome, reflecting a phenotypic evolution driven by the adaptation to the local environments or different breeding objectives [4].

With the development of high-throughput sequencing technology, such as chip technology, resequencing techniques, transcriptome sequencing, etc., and the assembly of the goat genome, research can be carried out at the genome level. Recently, several studies demonstrated hypoxia adaptation in Tibetan goat populations. In Tibetan cashmere goats from the Bange, Ritu, and Cuoqin counties, the *EPAS1* (Endothelial PAS domain protein 1) gene was shown to have a strong selection signal and a functional mutation [5]. The *DSG3* (Desmoglein 3) gene was also shown to contribute to high-altitude hypoxia adaptation of Tibetan cashmere goats [6]. In the Qinghai Tibetan goat, four genes (*CDK2* (Cyclin dependent kinase 2), *SOCS2* (Suppressor of cytokine signaling 2), *NOXA1* (NADPH oxidase activator 1) and *ENPEP* (Glutamyl aminopeptidase)) were identified as being important for hypoxia adaptation [7]. Interestingly, these results are inconsistent, indicating that Tibetan goats from different regions may have different mechanisms of high-altitude adaptation. 

In Nagqu, the Tibetan goat is found at elevations above 4500 m and is well protected by the local government. However, the mechanism of high-altitude adaptation in this breed is still unclear. The genetic structure of the Tibetan goat suggests that it belongs to the Northwest China population [8]. Therefore, we investigated the selection signal of hypoxic adaptation of Tibetan goats from Nagqu County. We genotyped four goat breeds using the Illumina Caprine 50K SNP (single nucleotide polymorphism) Chip. One highland breed (Tibetan goat, XZS) compared with three lowland breeds (Xinjiang goats, XJS; Taihang goats, THS; Huanghuai goats, HHS) to identify the selection signals of high-altitude adaptation [9]. Our findings can be used to better understand genomic signatures under selection, as well as increase understanding of the hypoxic adaptation mechanism of Tibetan goats on the plateau. 

## 2. Materials and Methods 

### 2.1. Biological Sample Collection and Genotyping

All experimental procedures involving goats were approved by the Science Research Department (in charge of animal welfare issue) of the Institute of Animal Sciences, Chinese Academy of Agricultural Sciences (IAS-CAAS) (Beijing, China). Ethical approval on animal survival was given by the animal ethics committee of IAS-CAAS (No. IASCAAS-AE-03, 12 December 2016). In this study, a total of four domestic goat breeds were included: Xinjiang goat, Tibetan goat, Huanghuai goat and Taihang goat. All of the samples were collected from conservation farms or core areas of origin (Table 1). All specimens were randomly selected. Genomic DNA samples were obtained by blood collection from the jugular vein and shipped to our laboratory for processing using the TIANamp Blood DNA Kit (Tiangen Biotech Co. Ltd., Beijing, China). We used a Nanodrop 2000 nucleic acid protein analyzer (Thermo, Waltham, MA, USA) to measure the purity and concentration of genomic DNA. All of these samples were genotyped using the Illumina Caprine 50K SNP Chip, which contains 53,347 SNPs.

### 2.2. Quality Control

To increase the accuracy of data processing, stringent quality control criteria were applied. SNPs were removed from the panel if the following four criteria were not met by PLINK 1.09: (i) Hardy–Weinberg equilibrium *p*-value less than 10^−4^; (ii) SNP call rate greater than 95%; (iii) single nucleotide polymorphism minimum allele frequency (MAF) greater than 0.05; (iv) the presence of mapped and autosomal loci [10].

### 2.3. Population Structure Analysis

The principal components analysis (PCA) identifies the principal components that represent the population structure based on genetic correlations between individuals. PCA was performed using PLINK1.09 [11]. The neighbor-joining tree (N-J tree) for individuals was also constructed using VCFtools [12] and Tassel [13]. ITOL software (https://itol.embl.de/upload.cgi) was used to visualize the N-J tree [14]. The N-J tree for populations was constructed using Split tree 4.0 [15]. To corroborate PCA and the N-J tree results, ADMIXTURE was implemented to reveal admixture patterns among breeds [16]. The K-value was set from 2 to 4. The solutions for each K-value were visualized using Excel 2010. 

### 2.4. Selection Signal Analyses

The genome regions with differentially fixed variants or strongly differing in allele frequency between separate breeds were identified by *Fst*, which was the conventional measure of population genetic differentiation. Matrix pairwise *Fst* values per SNP between breeds were estimated for all loci between populations using Genepop 4.2.2 software [17]. Briefly, *Fst* was estimated as follows:

(1) $$Fst\;=\;\frac{MSP-MSG}{MSP+\left(n_{c}-1\right)MSG}$$

where *MSG* is the mean square error within the population, *MSP* is the mean square error between the populations, and *n_c_* is the average sample size of the entire population after correction. This method is mainly applicable to the detection of selection signals between different populations and was originally used for the detection of selection signals between populations in human genetics [18]. In addition, the *Fst* values obtained for pairwise comparisons at each SNP were breed-standardized using *di*. The standardized *Fst* values were calculated (*di*) as:

*di* value = ∑_j≠i_[(*Fst*^ij^ − E[*Fst*^ij^])/sd[*Fst*^ij^]], (2)

where E[*Fst*^ij^] is the expected value and sd[*Fst*^ij^]] denotes the standard deviation of *Fst* between breeds i and j calculated from all analyzed SNP. 

### 2.5. Gene Annotation Analysis

Putatively selected loci were defined as genetic regions in overlapped SNPs with extremely high *di* values (top 1% level). Based on the goat reference genome annotation, the 50 kb upstream and downstream of significant *di* loci were operationally defined as candidate regions under selection. Gene function was determined using National Center for Biotechnology Information Gene (NCBI, http://www.ncbi.nlm.nih.gov/gene/) and a literature search. We selected candidate genes with high *di* value without the LOC (Locus) symbols and Gene IDs. Some genes related to high-altitude adaptation were used for functional enrichment analysis by Cytoscape software [19] and the ClueGO 2.2.0 plug-in [20], using Symbol ID as input parameters, the background organism selected *B. taurus*. *p* values < 0.05 after Bonferroni correction for multiple testing were considered statistically significant. Then, these genes were also used for analysis by DAVID 6.8 (https://david.ncifcrf.gov/) and Kobas 3.0 (http://kobas.cbi.pku.edu.cn/kobas3/genelist/) for Kyoto encyclopedia of genes and genomes (KEGG) analysis [21]. The background organism selected *Capra hircus*. *Q* values < 0.05 were considered statistically significant.

### 2.6. Validation of Selection Signal Results by q-PCR

#### 2.6.1. RNA Extraction

We also choose another six goats, containing three Tibetan goats (female, Nagqu Tibet) and three Huanghuai goats (female, Zhumadian, Henan, China). Heart and lung tissues were collected in these goats. Total RNA was separately isolated and purified using TRIzol (Invitrogen, Inc., Carlsbad, CA, USA). The extracted RNA was treated with RNase free DNase I (Ambion, Inc., Austin, TX, USA) to remove genomic DNA contamination. Additionally, we used a Nanodrop 2000 nucleic acid protein analyzer (Thermo, Waltham, MA, USA) to measure the purity and concentration of RNA.

#### 2.6.2. Reverse Transcription and q-PCR Analysis

Reverse transcription was conducted in accordance with the instructions of Prime Script II 1st strand kit (Takara, Dalian, China). cDNA could be used for downstream analysis. The primer of *LEPR* (leptin receptor), *EGFR* (epidermal growth factor receptor), *LDB1* (LIM domain binding 1), *NOX4* (NADPH oxidase 4) and *FGF2* (fibroblast growth factor 2) genes was designed by NCBI (https://www.ncbi.nlm.nih.gov/tools/primer-blast/index.cgi). β-actin was set as a reference gene. The experimental primer pairs for *LEPR*, *EGFR*, *LDB1, NOX4* and *FGF2* and β-actin are listed in Table S1. Three biological replicates for each breed were collected, and each performed in triplicate as technical replicates. The q-PCR reaction included one cycle of 95 °C for 2 min, followed by forty cycles of 95 °C for 5 s, 60 °C for 30 s and 72 °C for 10 s. Melting curves were analyzed to test the accuracy of the data. The results were used for the analysis of statistically significant differences in expression. All experimental data were analyzed by using equation 2^-ΔΔCt^. In addition, we used the ANOVA program in SPSS version 19.0 for statistical analysis. All statistical tests were considered statistically significant at *p* < 0.05. Plots were made using GraphPad Prism version 8.0.2 software (GraphPad Software, San Diego, CA, USA).

## 3. Results and Discussion

### 3.1. Genetic Variation and Population Genetic Analysis

**Figure 1:**
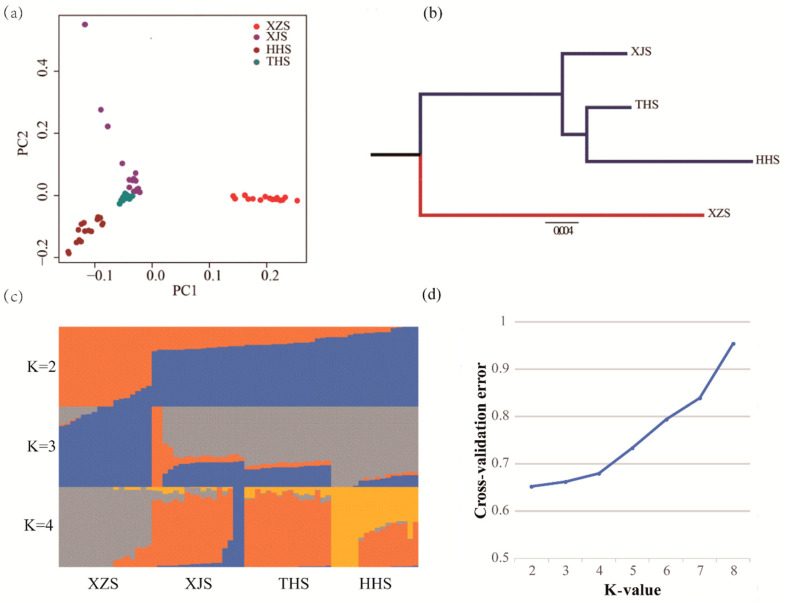
(**a**) The genetic relationship of all individuals. Symbols were used to distinguish Tibetan goats (XZS, red points), Xinjiang goats (XJS, purple points), Huanghuai goats (HHS, dark red points) and Taihang goats (THS, blue-green points). (**b**) Neighbor-joining (N-J) tree for the four breeds based on pairwise Fst. Low-altitude breeds are indicated in blue, and the high-altitude breed is indicated in red. (**c**) Model-based clustering of goat populations. Typical results are shown for K = 2–4. (**d**) When K = 2, the least amount of cross-validation error occurred.

After data quality control, 49,141 SNP markers were passed through filters and quality control. PCA showed two principal components (PC1 and PC2). By combining PC1 and PC2, all animals could be divided into two groups: low altitude population (XJS, HHS and THS) and high-altitude population (XZS) (Figure 1a). Analysis of breeding history of these four Chinese indigenous breeds confirmed the results of the PCA. The N-J tree for populations also showed that clearly defined clusters of XJS, HHS and THS were found in one main branch, and XZS was in another main branch (Figure 1b). To further examine the relationships among populations, we performed ADMIXTURE on all individuals. The number of populations varied from K = 2 to 4 (Figure 1c), and the least amount of cross-validation error occurred when K = 2, which makes it clear that K = 2 was the optimal modelling choice. Therefore, the four goat breeds could be appropriately divided into two groups. This result was consistent with the PCA and N-J tree (Figure 1d). These results are consistent with the breeding history. After quality control and population structure analysis, no outliers were detected; therefore, no animals were removed from further analysis.

All paired *Fst* values between the four breeds were calculated and adjusted to *Fst*/(1-*Fst*). It was found that the differentiation degree between THS and XJS was the lowest among all groups (0.016). The difference in *Fst* between XZS and HHS was the largest (0.058). In comparison, the average *Fst* of each breed was calculated more closely within the pedigree than between the pedigrees. The results showed that the *Fst* of THS was the lowest (0.029) and XZS was the highest (0.049) (Table 2). The pedigree of differentiation is consistent with N-J tree and ADMIXTURE.

### 3.2. Detecting Breed Positive Selection Genes 

**Figure 2:**
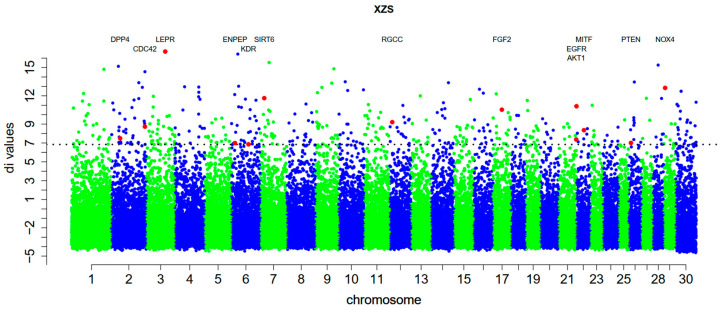
Overview of selection signals in the Tibetan goat plotted by *di* values. Some genes were highlighted by red plot.

To determine whether selection for adaptation to chronic hypoxia in Tibetan goat acts on recently occurring or pre-existing variation, we investigated, firstly, the population differentiation in Tibetan goat using *di* statistics based on the *Fst* value [22]. Secondly, the putatively selected loci were defined as genetic regions in overlapping SNPs with extremely high *di* values (top 1% level) (Figure 2). Thirdly, we obtained 261 specific selected SNPs that contained 459 genes without the LOC symbols and Gene IDs (Table S2). In accordance with gene function and previous study, finally, we found 16 potential genes associated with hypoxia and angiogenesis, energy metabolism, or melanogenesis [23,24,25]. 

**Figure 3:**
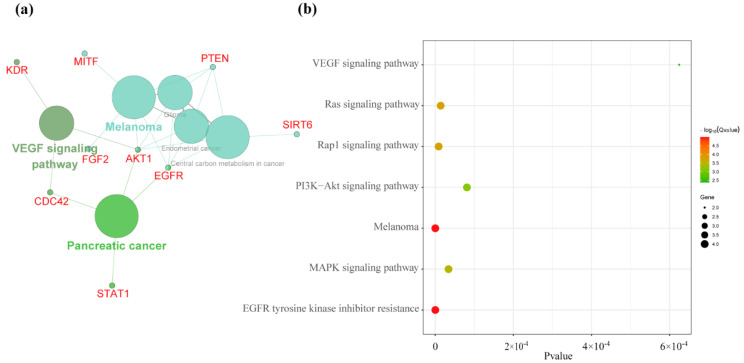
(**a**) Analysis of enrichment for the potential genes related to high-altitude adaptation by ClueGO; (**b**) Kyoto encyclopedia of genes and genomes (KEGG) analysis for the potential genes related to high-altitude adaptation by Kobas.

High-altitude adaptation may be caused by multiple genes that act in concert with one another [26]. Therefore, we performed ClueGO functional analysis and Kobas analysis on the potential genes and constructed a network of plausible pathways for high-altitude adaptation. ClueGO analysis demonstrated that most selected genes were regulated by the *VEGF* signaling pathway (*p* value _Bonferroni_ = 0.0015) and Melanoma (*p* value _Bonferroni_ = 0.000011) (Figure 3a). Kobas analysis also showed that most selected genes were enriched in Melanoma (*Q*-value = 1.01 × 10^−5^) and the VEGF signaling pathway (*Q*-value = 0.0042) (Figure 3b). Our results are similar to the Tibetan pig and Tibetan sheep [23,27] and indicate that different plateau animals have similar adaptive evolutionary characteristics. In addition, some cancer pathways were also identified in enrichment analysis, such as Pancreatic cancer (*p* value _Bonferroni_ = 1.6901 × 10^−4^), Glioma (*p* value _Bonferroni_ = 3.2299 × 10^−4^) and Endometrial cancer (*p* value _Bonferroni_ = 3.5066 × 10^−4^). Previous studies also have shown that oxygen homeostasis is essential for the maintenance of life and that perturbations of this process play an important role in the pathogenesis of a wide variety of diseases including cancer and inflammatory conditions [28,29]. Network analysis suggested that nine key genes (*FGF2, EGFR, AKT1, PTEN, MITF, ENPEP*, *SIRT6, KDR*, and *CDC42*) have important roles in the high-altitude adaptation of the Tibetan goat (Figure 3a).

Among these genes, some genes were detected in other species. First was the *ENPEP* gene (Glutamyl aminopeptidase) (*di* value = 7), which has biological functions associated with high-altitude adaptation and was also selected in the Tibetan sheep [23], Tibetan goat [7], and native Andeans [30]. Second was the *MITF* gene (melanocyte inducing transcription factor) (*di* value = 8.36), which was selected for adaptation to high levels of ultraviolet radiation in Tibetan goats. Similar results have been found for Tibetan sheep [23] and Ethiopian sheep [24]. Another, *RGCC* (regulator of cell cycle) (*di* value = 9.21) had a physical interaction with *HIF1α* and *VEGF*, which are key mediators in the cellular response to hypoxia [31] and also identify in Tibetan pigs [25]. 

The other newly detected genes were related to hypoxic adaptation, angiogenesis, and energy metabolism in Tibetan goats. The HIF-1 pathway is crucial for the hypoxic response in metazoans [32]. In the present study, it was induced and activated via *AKT1* (AKT serine/threonine kinase 1) (*di* value = 7.37) and *EGFR* (*di* value = 10.9) [33,34]. Similarly, *SIRT6* (Sirtuin 6) (*di* value = 11.75) can impede the necrosis/apoptosis pathways, leading to improved survival of cardiomyocytes following hypoxia [35]. 

Angiogenesis is one of the key mechanisms of high-altitude adaptation [36,37]. Remarkably, *FGF2* (fibroblast growth factor 2) (*di* value = 10.53), one of the fibroblast growth factor family members (*FGFs)* [38], has been proved as an angiogenic growth factor [39]. Furthermore, *CDC42* (cell division cycle 42) (*di* value = 8.73) mediates Bmp-induced sprouting angiogenesis [40], whilst *KDR* (kinase insert domain receptor) (*di* value = 6.86) attenuates *VEGF*-stimulated signaling to reduce angiogenesis under chronic hypoxia [41]. Selection for genes associated with energy metabolism is necessary for Tibetan goats to adapt to the cold environments they inhabit. We found that the *PTEN* (phosphatase and tensin homolog) (*di* value = 7) directly regulated glucose metabolism through dephosphorylation [42]. 

We also found that some strongly selected genes were related to high-altitude adaption in the Nagqu Tibetan goat. Remarkably, in chromosome 3, there was a strongly selected SNP involving the *LEPR* (*di* value = 16.70) gene, which has recently been demonstrated to induce upregulation of the hypoxic ventilatory response [43]. Comparative studies on altitude adaptability showed that the *LDB1* (*di* value = 13.47) gene on chromosome 26 was strongly selected. The nuclear adapter *LDB1* is the core component of a multiprotein transcription complex that regulates differentiation of multiple cell types, and plays an important role in regulating the transcription process responsible for maintaining hematopoietic stem cells [44]. Furthermore, *NOX4* (*di* value = 12.84), which contributes to hypoxia-induced angiogenesis by producing H_2_O_2_ [45], was strongly selected. In addition, *DPP4* (dipeptidyl peptidase 4) (*di* value = 7.5), an important enzyme involved in glycolysis and the maintenance of glucose stability [46], has also been detected in the Tibetan sheep [23]. 

It is noteworthy that Tibetan goat has other characteristics and traits besides plateau adaptability, such as slow growth, small body size and low fecundity. So, some relative genes were detected, the most special gene was *LEPR*, which not only related to hypoxic, but was also associated with reproductive and fattiness traits in goats and sheep [47,48]. *PROP1* (PROP paired-like homeobox 1) (*di* value = 15.53), another strongly selected gene, proved to be associated with growth traits of goat and sheep [49,50]. In addition, *MKLN1* (muskelin 1) (*di* value = 12.37) was associated with backfat thickness in Pigs [51] and *CRH* (corticotropin releasing hormone) (*di* value = 10.54) proved to be a candidate gene for body conformation traits in cows [52]. These findings have contributed to understanding the trace of domestication in Tibetan goats.

### 3.3. Validation of Selection Signal Analysis Results by q-PCR

**Figure 4:**
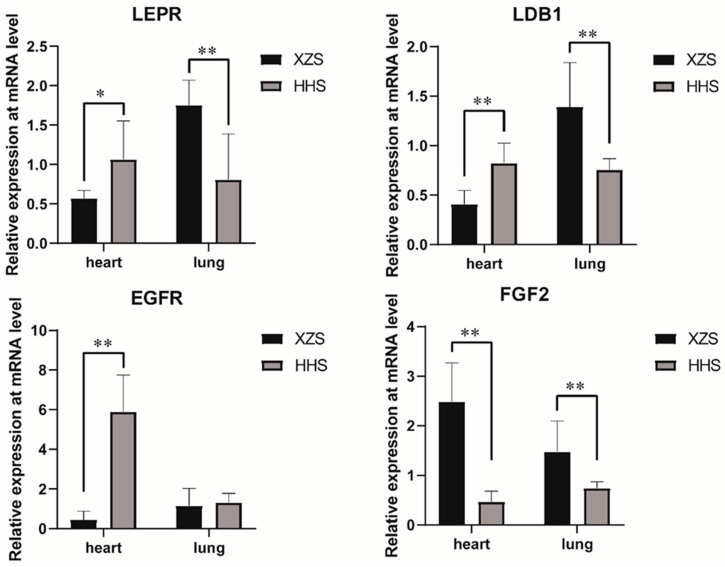
Expression levels of *LEPR*, *LDB1*, *EGFR* and *EGF2* in heart and lung tissues. * represents significant difference (*p <* 0.05) and ** represents extremely significant difference (*p <* 0.01).

In order to further validate the potential genes and detect the expression of genes in goats living in different altitudes, we chose five genes (*LEPR*, *LDB1*, *EGFR*, *NOX4* and *FGF2*) with high *di* value and detected their expression in the heart and lung by q-PCR (Figure 4). The result shows that *NOX4* gene expression was not different in heart and lung tissues of the two goat breeds. Expression levels of *LEPR*, *LDB1* and *FGF2* in the lungs of Tibetan goats were higher than in Huanghuai goats. *LEPR*, *EGFR* and *LDB1* had higher expression in the hearts of the Huanghuai goats than in Tibetan goats, but *FGF2* higher expression in the hearts of Tibetan goats than in Huanghuai goats (Figure 4). Our results suggest that differences in the expression of *LEPR*, *LDB1*, *EGFR* and *FGF2* genes may be related to the high-altitude adaptation, and these genes may be novel selection signals in goats. But there are unclear about these genes play a role in Tibetan sheep’s high-altitude adaptation mechanisms. Further study is also needed to determine how these genes work in Tibetan goats. 

## 4. Conclusions

Our study demonstrates that many high-altitude adaptive genes in the Nagqu Tibetan goats are specific to that breed. We found that *LEPR*, *LDB1*, *EGFR* and *EGF2* genes may play an impotent role in high-altitude adaption. Our findings improve the understanding of the selection of high-altitude adaptation in Tibetan goats and provide theoretical knowledge for the conservation and utilization of germplasm resources. Furthermore, they demonstrate the suitability of the Tibetan goat as a model for studying the mechanisms underlying adaptation to chronic hypoxia.

## Figures and Tables

**Table 1:** Information of the Chinese goat populations in this study.

Breeds	Abbr.	Sample Size	Sex	Breed Characteristics	Location	Living Altitude
Tibetan	XZS	17	female	multipurpose, horned, parti-color, small size	Naqu, Tibet, China	above 4500 m
Xinjiang	XJS	17	female	multipurpose, horned, white color, middle size	Tacheng, Xinjiang, China	about 1100 m
Taihang	THS	16	female	multipurpose, horned, black color, middle size	Changzhi, Shanxi, China	about 800 m
Huanghuai	HHS	16	female	meat, horned white color, big size	Zhumadian, Henan, China	about 81 m

**Table 2:** Fst/(1-Fst) values between pairs of four goat populations.

Breed	XZS	XJS	HHS	Mean Pairwise *Fst*
XZS				0.049
XJS	0.043			0.030
HHS	0.058	0.032		0.039
THS	0.044	0.016	0.026	0.029

## Supplementary Materials

The following are available online at https://www.mdpi.com/2076-2615/10/9/1599/s1, Table S1: Primers used in this study for q-PCR. Table S2: Putatively selected loci were defined as genetic regions in overlapped windows with extremely high di values (top 1% level).

## References

[1] Du L.X. (2011). Animal Genetic Resources in China.

[2] Thompson L.G., Yao T., Mosley-Thompson E., Davis M.E., Henderson K.A., Lin P. (2000). A high-resolution millennial record of the south asian monsoon from himalayan ice cores. Science.

[3] Deng J., Feng J., Li L., Zhong T., Wang L., Guo J., Ba G., Song T., Zhang H. (2018). Polymorphisms, differentiation, and phylogeny of 10 Tibetan goat populations inferred from mitochondrial D-loop sequences. Mitochondrial DNA A DNA Mapp. Seq. Anal..

[4] Guo J., Tao H., Li P., Li L., Zhong T., Wang L., Ma J., Chen X., Song T., Zhang H. (2018). Whole-genome sequencing reveals selection signatures associated with important traits in six goat breeds. Sci. Rep..

[5] Song S., Yao N., Yang M., Liu X., Dong K., Zhao Q., Pu Y., He X., Guan W., Yang N. (2016). Exome sequencing reveals genetic differentiation due to high-altitude adaptation in the Tibetan cashmere goat (*Capra hircus*). BMC Genom..

[6] Kumar C., Song S., Jiang L., He X., Zhao Q., Pu Y., Malhi K.K., Kamboh A.A., Ma Y. (2018). Sequence Characterization of DSG3 Gene to Know Its Role in High-Altitude Hypoxia Adaptation in the Chinese Cashmere Goat. Front. Genet..

[7] Wang X., Liu J., Zhou G., Guo J., Yan H., Niu Y., Li Y., Yuan C., Geng R., Lan X. (2016). Whole-genome sequencing of eight goat populations for the detection of selection signatures underlying production and adaptive traits. Sci. Rep..

[8] Wei C., Lu J., Xu L., Liu G., Wang Z., Zhao F., Zhang L., Han X., Du L., Liu C. (2014). Genetic structure of Chinese indigenous goats and the special geographical structure in the Southwest China as a geographic barrier driving the fragmentation of a large population. PLoS ONE.

[9] Kijas J.W., Ortiz J.S., McCulloch R., James A., Brice B., Swain B., Tosser-Klopp G., International Goat Genome C. (2013). Genetic diversity and investigation of polledness in divergent goat populations using 52 088 SNPs. Anim. Genet..

[10] Yuan Z., Liu E., Liu Z., Kijas J.W., Zhu C., Hu S., Ma X., Zhang L., Du L., Wang H. (2017). Selection signature analysis reveals genes associated with tail type in Chinese indigenous sheep. Anim. Genet..

[11] Purcell S., Neale B., Todd-Brown K., Thomas L., Ferreira M.A., Bender D., Maller J., Sklar P., de Bakker P.I., Daly M.J. (2007). PLINK: A tool set for whole-genome association and population-based linkage analyses. Am. J. Hum. Genet..

[12] Danecek P., Auton A., Abecasis G., Albers C.A., Banks E., DePristo M.A., Handsaker R.E., Lunter G., Marth G.T., Sherry S.T. (2011). The variant call format and VCFtools. Bioinformatics.

[13] Bradbury P.J., Zhang Z., Kroon D.E., Casstevens T.M., Ramdoss Y., Buckler E.S. (2007). TASSEL: Software for association mapping of complex traits in diverse samples. Bioinformatics.

[14] Letunic I., Bork P. (2019). Interactive Tree Of Life (iTOL) v4: Recent updates and new developments. Nucleic Acids Res..

[15] Huson D.H., Bryant D. (2006). Application of phylogenetic networks in evolutionary studies. Mol. Biol. Evol..

[16] Alexander D.H., Novembre J., Lange K. (2009). Fast model-based estimation of ancestry in unrelated individuals. Genome Res..

[17] Rousset M.R.F. (1995). Population genetics software for exact tests and ecumenicism. J. Hered..

[18] Akey J.M., Zhang G., Zhang K., Jin L., Shriver M.D. (2002). Interrogating a high-density SNP map for signatures of natural selection. Genome Res..

[19] Saito R., Smoot M.E., Ono K., Ruscheinski J., Wang P.-L., Lotia S., Pico A.R., Bader G.D., Ideker T. (2012). A travel guide to Cytoscape plugins. Nat. Methods.

[20] Bindea G., Mlecnik B., Hackl H., Charoentong P., Tosolini M., Kirilovsky A., Fridman W.-H., Pagès F., Trajanoski Z., Galon J. (2009). ClueGO: A Cytoscape plug-in to decipher functionally grouped gene ontology and pathway annotation networks. Bioinformatics.

[21] Huang D.W., Sherman B.T., Lempicki R.A. (2009). Systematic and integrative analysis of large gene lists using DAVID bioinformatics resources. Nat. Protoc..

[22] Akey J.M., Ruhe A.L., Akey D.T., Wong A.K., Connelly C.F., Madeoy J., Nicholas T.J., Neff M.W. (2010). Tracking footprints of artificial selection in the dog genome. Proc. Natl. Acad. Sci. USA.

[23] Wei C., Wang H., Liu G., Zhao F., Kijas J.W., Ma Y., Lu J., Zhang L., Cao J., Wu M. (2016). Genome-wide analysis reveals adaptation to high altitudes in Tibetan sheep. Sci. Rep..

[24] Edea Z., Dadi H., Dessie T., Kim K.S. (2019). Genomic signatures of high-altitude adaptation in Ethiopian sheep populations. Genes Genom..

[25] Ai H., Yang B., Li J., Xie X., Chen H., Ren J. (2014). Population history and genomic signatures for high-altitude adaptation in Tibetan pigs. BMC Genom..

[26] Bigham A.W., Lee F.S. (2014). Human high-altitude adaptation: Forward genetics meets the HIF pathway. Genes Dev..

[27] Zhang B., Chamba Y., Shang P., Wang Z., Ma J., Wang L., Zhang H. (2017). Comparative transcriptomic and proteomic analyses provide insights into the key genes involved in high-altitude adaptation in the Tibetan pig. Sci. Rep..

[28] Macklin P.S., McAuliffe J., Pugh C.W., Yamamoto A. (2017). Hypoxia and HIF pathway in cancer and the placenta. Placenta.

[29] Huang D., Li C., Zhang H. (2014). Hypoxia and cancer cell metabolism. Acta Biochim. Biophys. Sin..

[30] Eichstaedt C.A., Antao T., Cardona A., Pagani L., Kivisild T., Mormina M. (2015). Genetic and phenotypic differentiation of an Andean intermediate altitude population. Physiol. Rep..

[31] An X., Jin Y., Guo H., Foo S.Y., Cully B.L., Wu J., Zeng H., Rosenzweig A., Li J. (2009). Response gene to complement 32, a novel hypoxia-regulated angiogenic inhibitor. Circulation.

[32] Lendahl U., Lee K.L., Yang H., Poellinger L. (2009). Generating specificity and diversity in the transcriptional response to hypoxia. Nat. Rev. Genet..

[33] Lee S.H., Koo K.H., Park J.W., Kim H.J., Ye S.K., Park J.B., Park B.K., Kim Y.N. (2009). HIF-1 is induced via EGFR activation and mediates resistance to anoikis-like cell death under lipid rafts/caveolae-disrupting stress. Carcinogenesis.

[34] Pez F., Dayan F., Durivault J., Kaniewski B., Aimond G., Le Provost G.S., Deux B., Clézardin P., Sommer P., Pouysségur J. (2011). The HIF-1–inducible lysyl oxidase activates HIF-1 via the Akt pathway in a positive regulation loop and synergizes with HIF-1 in promoting tumor cell growth. Cancer Res..

[35] Wang X.-X., Wang X.-L., Tong M.-M., Gan L., Chen H., Wu S.-S., Chen J.-X., Li R.-L., Wu Y., Zhang H.-Y. (2016). SIRT6 protects cardiomyocytes against ischemia/reperfusion injury by augmenting FoxO3α-dependent antioxidant defense mechanisms. Basic Res. Cardiol..

[36] Gassmann N.N., van Elteren H.A., Goos T.G., Morales C.R., Rivera-Ch M., Martin D.S., Cabala Peralta P., Passano Del Carpio A., Aranibar Machaca S., Huicho L. (2016). Pregnancy at high altitude in the Andes leads to increased total vessel density in healthy newborns. J. Appl. Physiol..

[37] Koester-Hegmann C., Bengoetxea H., Kosenkov D., Thiersch M., Haider T., Gassmann M., Schneider Gasser E.M. (2018). High-Altitude Cognitive Impairment Is Prevented by Enriched Environment Including Exercise via VEGF Signaling. Front. Cell. Neurosci..

[38] Brem H., Klagsbrun M. (1992). The role of fibroblast growth factors and related oncogenes in tumor growth. Cancer Treat. Res..

[39] Presta M., Andrés G., Leali D., Dell’Era P., Ronca R.J.E.C.N. (2009). Inflammatory cells and chemokines sustain FGF2-induced angiogenesis. Eur. Cytokine Netw..

[40] Wakayama Y., Fukuhara S., Ando K., Matsuda M., Mochizuki N. (2015). Cdc42 mediates Bmp-induced sprouting angiogenesis through Fmnl3-driven assembly of endothelial filopodia in zebrafish. Dev. Cell.

[41] Olszewska-Pazdrak B., Hein T.W., Olszewska P., Carney D.H. (2009). Chronic hypoxia attenuates VEGF signaling and angiogenic responses by downregulation of KDR in human endothelial cells. Am. J. Physiol. Cell Physiol..

[42] Qian X., Li X., Shi Z., Xia Y., Cai Q., Xu D., Tan L., Du L., Zheng Y., Zhao D. (2019). PTEN Suppresses Glycolysis by Dephosphorylating and Inhibiting Autophosphorylated PGK1. Mol. Cell.

[43] Caballero Eraso C., Shin M., Pho H., Schwartz A., Tang W.-Y., Sham J., Polotsky V. (2018). Mechanisms and Significance of Leptin-Induced Upregulation of the Hypoxic Ventilatory Response. D29. Pathophysiology of OSA: Intermittent Hypoxia and Beyond.

[44] Li L., Jothi R., Cui K., Lee J.Y., Cohen T., Gorivodsky M., Tzchori I., Zhao Y., Hayes S.M., Bresnick E.H. (2011). Nuclear adaptor Ldb1 regulates a transcriptional program essential for the maintenance of hematopoietic stem cells. Nat. Immunol..

[45] Zhu Y., Ni T., Lin J., Zhang C., Zheng L., Luo M. (2019). Long non-coding RNA H19, a negative regulator of microRNA-148b-3p, participates in hypoxia stress in human hepatic sinusoidal endothelial cells via NOX4 and eNOS/NO signaling. Biochimie.

[46] Das S.S., Hayashi H., Sato T., Yamada R., Hiratsuka M., Hirasawa N. (2014). Regulation of dipeptidyl peptidase 4 production in adipocytes by glucose. Diabetes Metab. Syndr. Obes..

[47] Gunawan A., Pramukti F., Listyarini K., Abuzahra M., Jakaria C.S., Inounu I., Uddin M. (2019). Novel variant in the leptin receptor (LEPR) gene and its association with fat quality, odour, and flavour in sheep. JITAA.

[48] Alim M., Hossain M., Nusrat J., Salimullah M., Shu-Hong Z., Alam J. (2019). Genetic effects of leptin receptor (LEPR) polymorphism on litter size in a Black Bengal goat population. Anim. Biol..

[49] Ekegbu U.J., Burrows L., Amirpour-Najafabadi H., Zhou H., Hickford J.G. (2019). Gene polymorphisms in PROP1 associated with growth traits in sheep. Gene.

[50] Ma L., Qin Q., Yang Q., Zhang M., Zhao H., Pan C., Lei C., Chen H., Lan X. (2017). Associations of six SNPs of POU1F1-PROP1-PITX1-SIX3 pathway genes with growth traits in two Chinese indigenous goat breeds. Ann. Anim. Sci..

[51] Lee Y.-S., Shin D. (2018). Genome-Wide Association Studies Associated with Backfat Thickness in Landrace and Yorkshire Pigs. Genom. Inform..

[52] Kowalewska-Luczak I., Czerniawska-Piatkowska E., Kowalczyk A. (2019). Relationship between polymorphism in the CRH gene and the traits of body conformation of Salers cows. Anim. Biotechnol..

