# Supplementary material for: Selection Signatures Analysis Reveals Genes Associated with High-Altitude Adaptation in Tibetan Goats from Nagqu, Tibet
Source: Animals (Basel). 2020 Sep 8;10(9):1599. doi: 10.3390/ani10091599 (PMC7552128; doi:10.3390/ani10091599)
Supplement: Supplementary file 1 [file animals-10-01599-s001.zip › Supplement Table/Table S1.pdf]

ID	position	chr	start	end	di	Gene
snp54289-scaffold83-525657	76209578	3	76162714	76317920	16.70	LEPR
snp17727-scaffold184-861468	25037421	10	24935999	25122183	13.49	AVEN
snp17727-scaffold184-861468	25037421	10	24921289	24999162	13.49	CHRM5
snp41102-scaffold532-545458	21185062	26	21228334	21231762	13.47	ELOVL3
snp41102-scaffold532-545458	21185062	26	21141754	21148842	13.47	LDB1
snp41102-scaffold532-545458	21185062	26	21182333	21192773	13.47	NOLC1
snp41102-scaffold532-545458	21185062	26	21233421	21243176	13.47	PITX3
snp41102-scaffold532-545458	21185062	26	21166732	21180872	13.47	PPRC1
snp7379-scaffold127-22944	106846311	2	106827675	106851734	13.39	ASIC4
snp7379-scaffold127-22944	106846311	2	106851902	106856866	13.39	CHPF
snp7379-scaffold127-22944	106846311	2	106814192	106821504	13.39	GMPPA
snp7379-scaffold127-22944	106846311	2	106887841	106890949	13.39	INHA
snp7379-scaffold127-22944	106846311	2	106865331	106886119	13.39	OBSL1
snp7379-scaffold127-22944	106846311	2	106859385	106865197	13.39	TMEM198
snp2518-scaffold1071-477188	119890175	2	119917739	119953380	12.93	TRIM62
snp59584-scaffold983-609280	5464673	29	5251872	5432690	12.84	NOX4
snp59584-scaffold983-609280	5464673	29	5461794	5571971	12.84	TYR
snp11512-scaffold1420-40982	96955132	10	96802259	97137189	12.64	FOXN3
snp33758-scaffold397-1371358	34739282	10	34752400	34796566	12.56	TMEM62
snp33758-scaffold397-1371358	34739282	10	34565526	34725689	12.56	UBR1
snp45884-scaffold63-3008636	30996340	30	30970998	31281222	12.48	GRIA3
snp32100-scaffold362-811160	92616687	4	92330009	92709988	12.38	MKLN1
snp50457-scaffold723-196705	7254399	9	7081335	7344049	12.33	BCKDHB
snp15414-scaffold1634-488849	37514796	13	37508636	37860922	11.99	SLC24A3
snp47935-scaffold675-2898965	28133226	3	28083566	28146683	11.95	LRIG2
snp32084-scaffold362-134195	91939722	4	91769318	91909928	11.91	COPG2
snp55237-scaffold85-2363783	16291240	7	16265763	16282966	11.75	ANKRD24
snp55237-scaffold85-2363783	16291240	7	16243124	16257270	11.75	CCDC94
snp55237-scaffold85-2363783	16291240	7	16305170	16316543	11.75	CREB3L3
snp55237-scaffold85-2363783	16291240	7	16259440	16265334	11.75	EBI3
snp55237-scaffold85-2363783	16291240	7	16296363	16304502	11.75	SIRT6
snp26248-scaffold275-1191080	18337373	27	18289158	18414979	11.74	MTUS1
snp27104-scaffold289-2210159	56266918	6	56259126	56330604	11.65	FAM114A1
snp27104-scaffold289-2210159	56266918	6	56234130	56257032	11.65	TLR6
snp985-scaffold1025-3046389	97443273	6	97387763	97953980	11.53	ARHGAP24
snp25796-scaffold266-695146	124797258	1	124721148	124761967	11.44	GRK7
snp25796-scaffold266-695146	124797258	1	124796798	124803873	11.44	RNF7
snp41241-scaffold535-546074	47768536	14	47774560	47807404	11.27	LY96
snp41241-scaffold535-546074	47768536	14	47733734	47754308	11.27	TCEB1
snp41241-scaffold535-546074	47768536	14	47761873	47768084	11.27	TMEM70
snp20922-scaffold2050-85852	5801467	2	5829532	5845614	11.24	NEMP2
snp10545-scaffold1376-836918	73764143	8	73760144	73825534	11.13	UBAP1
snp57542-scaffold92-3437740	7154989	30	7061109	7183789	11.10	PCDH19
snp21697-scaffold2121-649938	68267325	1	68159689	68302684	11.06	ZNF148
snp17157-scaffold1790-196441	7168075	23	7145089	7146070	11.00	B3GALT4
snp17157-scaffold1790-196441	7168075	23	7156247	7157540	11.00	PFDN6
snp17157-scaffold1790-196441	7168075	23	7158086	7164475	11.00	RGL2
snp17157-scaffold1790-196441	7168075	23	7138898	7143599	11.00	RPS18
snp17157-scaffold1790-196441	7168075	23	7167142	7177208	11.00	TAPBP
snp17157-scaffold1790-196441	7168075	23	7122854	7138733	11.00	VPS52
snp17157-scaffold1790-196441	7168075	23	7146777	7155932	11.00	WDR46
snp17157-scaffold1790-196441	7168075	23	7177290	7180090	11.00	ZBTB22
snp36027-scaffold431-5387495	50789168	12	50651317	50757797	10.99	SCEL
snp36027-scaffold431-5387495	50789168	12	50788052	50839350	10.99	SLAIN1
snp56584-scaffold894-1377437	13428714	30	13307355	13389021	10.93	FRMPD3

snp56584-scaffold894-1377437	13428714	30	13407960	13434049	10.93	PRPS1
snp6892-scaffold1251-915917	736907	22	785628	852401	10.90	EGFR
snp6892-scaffold1251-915917	736907	22	584063	747522	10.90	LANCL2
snp47921-scaffold675-2270475	27504736	3	27536095	27619801	10.80	PHTF1
snp47921-scaffold675-2270475	27504736	3	27430646	27489332	10.80	PTPN22
snp47921-scaffold675-2270475	27504736	3	27490950	27533531	10.80	RSBN1
snp52787-scaffold791-1202632	53852339	14	53784986	53840413	10.64	TPD52
snp51156-scaffold75-97384	41026652	14	41072834	41075167	10.55	CRH
snp51156-scaffold75-97384	41026652	14	40907431	41005497	10.55	DNAJC5B
snp51156-scaffold75-97384	41026652	14	41013233	41071860	10.55	TRIM55
snp24469-scaffold248-1981827	75342927	6	74809035	75749184	10.53	ADGRL3
snp43564-scaffold581-1449908	34655099	17	34698642	34711437	10.53	BBS12
snp43564-scaffold581-1449908	34655099	17	34572732	34623101	10.53	FGF2
snp33101-scaffold386-813440	34104967	7	34140493	34149120	10.49	ARL10
snp33101-scaffold386-813440	34104967	7	33904798	34081773	10.49	COMMD10
snp33101-scaffold386-813440	34104967	7	34150928	34155702	10.49	NOP16
snp55438-scaffold859-502903	8597199	19	8592630	8604524	10.44	MKS1
snp1265-scaffold1032-527606	40038557	7	40052356	40065398	10.36	APC2
snp1265-scaffold1032-527606	40038557	7	40068635	40073570	10.36	C7H19orf25
snp1265-scaffold1032-527606	40038557	7	40021283	40033073	10.36	DAZAP1
snp1265-scaffold1032-527606	40038557	7	40002559	40006626	10.36	GAMT
snp1265-scaffold1032-527606	40038557	7	39995886	40001859	10.36	NDUFS7
snp1265-scaffold1032-527606	40038557	7	40079120	40086271	10.36	PCSK4
snp1265-scaffold1032-527606	40038557	7	40086565	40091478	10.36	REEP6
snp1265-scaffold1032-527606	40038557	7	40033646	40038261	10.36	RPS15
snp17939-scaffold185-5714191	67344973	11	67377685	67422014	10.25	C11H2orf42
snp17939-scaffold185-5714191	67344973	11	67316495	67317969	10.25	PCBP1
snp44957-scaffold612-2110770	24401411	14	24356966	24360773	10.23	FAM84B
snp5961-scaffold121-842233	60191126	17	60233187	60354722	10.22	RBM19
snp28572-scaffold306-590080	62759584	1	62636308	62757367	10.12	ARHGAP31
snp28572-scaffold306-590080	62759584	1	62796734	62824745	10.12	POGLUT1
snp28572-scaffold306-590080	62759584	1	62763357	62792615	10.12	TMEM39A
snp32085-scaffold362-167849	91973376	4	91997852	91998275	10.06	KLF14
snp32085-scaffold362-167849	91973376	4	91911477	91940810	10.06	TSGA13
snp23237-scaffold2321-8304	13032718	7	13036630	13040242	10.02	MCEMP1
snp23237-scaffold2321-8304	13032718	7	12992845	12994405	10.02	PCP2
snp23237-scaffold2321-8304	13032718	7	12991004	12992856	10.02	PET100
snp23237-scaffold2321-8304	13032718	7	13027692	13029076	10.02	RETN
snp23237-scaffold2321-8304	13032718	7	13000329	13007750	10.02	STXBP2
snp23237-scaffold2321-8304	13032718	7	13042227	13043120	10.02	TRAPPC5
snp23237-scaffold2321-8304	13032718	7	12984937	12990947	10.02	XAB2
snp3244-scaffold1098-457580	4502046	11	4528687	4601617	9.91	EIF5B
snp3244-scaffold1098-457580	4502046	11	4466544	4475771	9.91	LYG1
snp3244-scaffold1098-457580	4502046	11	4515791	4527738	9.91	TXNDC9
snp27492-scaffold293-907201	11926983	11	11476305	12179043	9.90	EXOC6B
snp604-scaffold1017-348078	56400985	1	56347414	56363551	9.89	C1H3orf17
snp35336-scaffold424-186719	43969076	14	44012517	44155788	9.89	SLCO5A1
snp35336-scaffold424-186719	43969076	14	43808688	44000983	9.89	SULF1
snp9887-scaffold1352-1895771	92501014	6	92171959	92874079	9.83	C6H4orf22
snp23727-scaffold2397-129302	25168211	3	25157873	25187546	9.82	MAB21L3
snp25656-scaffold264-2485279	67062398	17	66957574	67019454	9.73	PITPNB
snp41145-scaffold532-2362038	19368482	26	19317143	19345584	9.68	CPN1
snp15377-scaffold1630-177736	58443867	11	57514933	58504409	9.66	LRRTM4
snp40394-scaffold515-4095049	90582403	2	90534869	90586090	9.64	CARF
snp40394-scaffold515-4095049	90582403	2	90585227	90772748	9.64	NBEAL1
snp40394-scaffold515-4095049	90582403	2	90508437	90534459	9.64	WDR12

snp32947-scaffold383-159900	69928227	5	69663588	70119857	9.63	LARGE
snp52786-scaffold791-1170476	53820183	14	53677377	53780299	9.63	MRPS28
snp57366-scaffold913-1308916	7929102	20	7650142	7928735	9.57	ARHGEF28
snp51555-scaffold757-135443	25866321	30	25748125	26122733	9.55	HS6ST2
snp33594-scaffold394-2924580	44645507	5	44622695	44636968	9.53	DYRK2
snp25660-scaffold264-2701728	67278847	17	67078521	67632659	9.50	TTC28
snp16421-scaffold173-1073466	3385427	16	3331255	3580861	9.45	SRGAP2
snp50762-scaffold734-1162323	24813881	25	24830748	24855485	9.45	KDM8
snp50762-scaffold734-1162323	24813881	25	24855559	24891405	9.45	NSMCE1
snp59904-scaffold997-1140734	75707948	30	75721825	75927494	9.43	AR
snp55187-scaffold849-159480	61789330	13	61811646	61908881	9.42	ITCH
snp9212-scaffold1336-68580	72963915	12	72933642	73220760	9.42	NALCN
snp41142-scaffold532-2260226	19470294	26	19518948	19527580	9.41	BLOC1S2
snp41142-scaffold532-2260226	19470294	26	19446514	19482778	9.41	CHUK
snp41142-scaffold532-2260226	19470294	26	19490160	19514644	9.41	CWF19L1
snp41142-scaffold532-2260226	19470294	26	19403189	19441221	9.41	ERLIN1
snp29609-scaffold321-1675691	48808361	3	48778655	48868913	9.39	KIAA1107
snp32259-scaffold366-1926353	83700229	8	83644455	83679264	9.38	SPATA31C2
snp2777-scaffold1080-70178	98775438	6	98596874	98816491	9.37	PTPN13
snp14196-scaffold1568-335289	51105459	1	50890041	51179306	9.32	BBX
snp8284-scaffold130-1125304	126962003	2	126920548	126926696	9.32	SYF2
snp7273-scaffold1269-654749	53250483	7	53275919	53294648	9.22	GRXCR2
snp7273-scaffold1269-654749	53250483	7	53149598	53232190	9.22	PRELID2
snp52831-scaffold792-330324	9553507	12	9528182	9530477	9.21	RGCC
snp52831-scaffold792-330324	9553507	12	9594837	9956871	9.21	VWA8
snp4582-scaffold1147-469297	72137334	30	72187276	72189276	9.19	CXHXorf65
snp4582-scaffold1147-469297	72137334	30	72183139	72186797	9.19	IL2RG
snp4582-scaffold1147-469297	72137334	30	72155265	72176855	9.19	MED12
snp4582-scaffold1147-469297	72137334	30	72130305	72151017	9.19	NLGN3
snp41103-scaffold532-580629	21149891	26	21114878	21117471	9.14	HPS6
snp40753-scaffold521-2252025	29398586	5	29428487	29431060	9.13	C1QL4
snp40753-scaffold521-2252025	29398586	5	29414012	29417381	9.13	DNAJC22
snp40753-scaffold521-2252025	29398586	5	29432200	29439635	9.13	TROAP
snp14894-scaffold16-2229656	8445993	8	8465609	8481346	9.12	PRSS55
snp14894-scaffold16-2229656	8445993	8	8398220	8445786	9.12	RP1L1
snp21065-scaffold2061-608263	8792700	9	8769596	8775683	9.08	FAM46A
snp49813-scaffold711-1595891	19692854	20	18950997	20290853	9.07	PDE4D
snp8318-scaffold130-2544343	128381042	2	128385560	128389278	9.07	RPL11
snp8318-scaffold130-2544343	128381042	2	128334448	128344948	9.07	TCEB3
snp33091-scaffold386-396847	33688374	7	33614278	33743021	9.04	SEMA6A
snp52988-scaffold796-515837	28808847	29	28623621	29234673	9.03	KIRREL3
snp54991-scaffold841-747978	14600266	3	14587287	14596108	9.03	ADAM15
snp54991-scaffold841-747978	14600266	3	14597814	14613863	9.03	DCST1
snp54991-scaffold841-747978	14600266	3	14614171	14626762	9.03	DCST2
snp54991-scaffold841-747978	14600266	3	14561935	14565030	9.03	EFNA3
snp54991-scaffold841-747978	14600266	3	14581511	14586071	9.03	EFNA4
snp54991-scaffold841-747978	14600266	3	14626843	14637296	9.03	ZBTB7B
snp29615-scaffold321-1976019	48508033	3	48251606	48474506	9.02	EVI5
snp29615-scaffold321-1976019	48508033	3	48498587	48501155	9.02	GFI1
snp45284-scaffold618-2195419	19114049	11	19083404	19153691	9.00	STRN
snp43749-scaffold588-774844	13831096	9	13527116	14710238	8.94	NKAIN2
snp24537-scaffold2495-85440	55477443	1	55483123	55519262	8.94	C1H3orf52
snp24537-scaffold2495-85440	55477443	1	55526538	55543775	8.94	GCSAM
snp24537-scaffold2495-85440	55477443	1	55427872	55473103	8.94	TMPRSS7
snp3666-scaffold112-341273	58759049	16	58791035	58919952	8.94	CEP350
snp3666-scaffold112-341273	58759049	16	58677896	58729856	8.94	TOR1AIP1

snp54733-scaffold837-2429935	54471875	15	54364463	54437469	8.88	BBOX1
snp9204-scaffold1335-579872	44445600	19	44409898	44430865	8.81	GOSR2
snp9204-scaffold1335-579872	44445600	19	44468551	44491768	8.81	WNT9B
snp11895-scaffold144-2363622	69290450	3	69335422	70349598	8.76	NEGR1
snp46578-scaffold646-549778	96502919	4	96339723	96487711	8.76	CNOT4
snp46578-scaffold646-549778	96502919	4	96533891	96611277	8.76	NUP205
snp46578-scaffold646-549778	96502919	4	96522172	96523032	8.76	TPT1
snp8353-scaffold130-4097690	129934389	2	129926618	129945931	8.73	CDC42
snp8353-scaffold130-4097690	129934389	2	129890982	129904623	8.73	WNT4
snp18546-scaffold1873-188325	1478448	12	1088626	1608949	8.72	DIAPH3
snp20370-scaffold202-255496	30032239	2	29931506	30143448	8.68	CSRNP3
snp40422-scaffold516-813964	72998924	15	72969518	73065742	8.68	CKAP5
snp40422-scaffold516-813964	72998924	15	72951348	72966477	8.68	F2
snp51480-scaffold754-1977841	19338874	21	19344168	19413812	8.68	ZNF710
snp17847-scaffold185-1738069	71321095	11	71318175	71323139	8.67	ATRAID
snp17847-scaffold185-1738069	71321095	11	71296208	71317540	8.67	CAD
snp17847-scaffold185-1738069	71321095	11	71283415	71287928	8.67	SLC30A3
snp17847-scaffold185-1738069	71321095	11	71323173	71335854	8.67	SLC5A6
snp58825-scaffold960-1227200	58832432	24	58845185	58847241	8.67	MC4R
snp2346-scaffold107-2452613	77478886	5	77477953	77568181	8.67	OVCH1
snp2346-scaffold107-2452613	77478886	5	77180226	77469882	8.67	TMTC1
snp40832-scaffold524-1556971	26599848	9	26538432	26560669	8.67	AMD1
snp40832-scaffold524-1556971	26599848	9	26593874	26763572	8.67	CDK19
snp38800-scaffold492-1591907	39785999	21	39596313	39830998	8.67	NUBPL
snp3966-scaffold1125-804912	38632289	16	38676076	38705654	8.60	AADACL4
snp43972-scaffold595-3101875	92193503	3	91824967	92309613	8.57	FAF1
snp21760-scaffold2131-1573314	48961677	19	48885727	49030322	8.57	TBCD
snp21760-scaffold2131-1573314	48961677	19	48971010	48979922	8.57	ZNF750
snp1704-scaffold1048-413128	49745828	15	49709708	49723304	8.56	PLEKHB1
snp1704-scaffold1048-413128	49745828	15	49737187	49776075	8.56	RAB6A
snp33637-scaffold395-633966	14197109	11	14133389	14182817	8.55	SRD5A2
snp17952-scaffold185-6260329	66798835	11	66741477	66902378	8.54	AAK1
snp54248-scaffold827-4346234	45388101	22	44386916	45346669	8.52	ERC2
snp54248-scaffold827-4346234	45388101	22	45371355	45381514	8.52	WNT5A
snp17493-scaffold181-148816	96493936	3	96509589	96686341	8.52	MAST2
snp29672-scaffold321-4646523	45837529	3	45865966	45876916	8.46	RWDD3
snp57641-scaffold923-1312864	55028971	2	53950365	56134849	8.45	LRP1B
snp25787-scaffold266-321974	125170430	1	125060875	125186297	8.42	ZBTB38
snp4851-scaffold1162-198452	25628222	15	25676078	25702241	8.41	BUD13
snp16222-scaffold1704-1001411	102628534	3	102666208	102672931	8.40	HEYL
snp16222-scaffold1704-1001411	102628534	3	102607335	102613340	8.40	HPCAL4
snp16222-scaffold1704-1001411	102628534	3	102618944	102636840	8.40	NT5C1A
snp55126-scaffold847-612212	7470934	26	7515220	7608644	8.40	A1CF
snp25897-scaffold2678-81588	14888953	30	14834131	14983701	8.40	GUCY2F
snp5951-scaffold121-413706	60619653	17	60594506	60598837	8.36	LHX5
snp5951-scaffold121-413706	60619653	17	60653198	60669019	8.36	PLBD2
snp5951-scaffold121-413706	60619653	17	60642623	60650656	8.36	SDS
snp5951-scaffold121-413706	60619653	17	60615084	60627393	8.36	SDSL
snp13853-scaffold154-2559968	31452643	22	31427864	31659079	8.36	MITF
snp34005-scaffold40-2680622	50848175	20	50332461	51128671	8.35	CDH12
snp3307-scaffold1101-219587	53629883	19	53534007	53581400	8.34	SEC14L1
snp59356-scaffold978-358352	27683765	8	27526581	27870426	8.29	CCDC171
snp8984-scaffold1327-407058	22074707	16	22084481	22089216	8.27	HLX
snp11339-scaffold141-716187	41486393	16	41511644	41526022	8.27	TMEM201
snp17038-scaffold1776-992575	66147024	16	66004442	66237103	8.26	KCNK2
snp28450-scaffold303-3829345	33351681	19	33277488	33333839	8.25	EPN2

snp28450-scaffold303-3829345	33351681	19	33389814	33419063	8.25	FAM83G
snp28450-scaffold303-3829345	33351681	19	33352697	33373867	8.25	GRAP
snp28450-scaffold303-3829345	33351681	19	33373894	33429939	8.25	SLC5A10
snp4606-scaffold1147-1932056	73600093	30	73567629	73594109	8.25	FAM155B
snp3089-scaffold1095-70867	31960895	12	31978856	32038252	8.21	SPATA13
snp25386-scaffold262-821529	22948497	20	22939592	23050756	8.14	ANKRD55
snp58106-scaffold94-6000063	68898128	6	68923448	68939086	8.11	SRD5A3
snp48086-scaffold68-3492027	48120058	10	47894820	48080535	8.07	LIPC
snp22502-scaffold2226-313730	14970953	14	14974398	15243512	8.07	FAM135B
snp32649-scaffold375-1025987	14251005	17	14153731	14279787	8.07	GAB1
snp2787-scaffold1080-574611	99279871	6	99304745	99321823	8.06	HSD17B13
snp11539-scaffold1421-449266	16706469	10	16644018	16695830	8.06	TLE3
snp44757-scaffold609-1219218	50829477	17	50810012	51017724	8.04	NCOR2
snp6380-scaffold1224-2308780	3956878	22	3625031	4580147	8.03	RBMS3
snp30665-scaffold339-4546335	45947673	7	45884093	45919583	7.99	CDC25C
snp30665-scaffold339-4546335	45947673	7	45924543	45934561	7.99	FAM53C
snp30665-scaffold339-4546335	45947673	7	45931976	45996880	7.99	KDM3B
snp10557-scaffold1376-1305714	73295347	8	73326383	73343421	7.96	AQP7
snp10557-scaffold1376-1305714	73295347	8	73250799	73316071	7.96	NFX1
snp546-scaffold1015-982216	41553201	28	41519177	41526270	7.95	MSMB
snp546-scaffold1015-982216	41553201	28	41495924	41517898	7.95	NCOA4
snp54194-scaffold827-1877476	42919343	22	42847895	42915664	7.94	ABHD6
snp54194-scaffold827-1877476	42919343	22	42916787	42937310	7.94	DNASE1L3
snp54194-scaffold827-1877476	42919343	22	42955805	43095189	7.94	FLNB
snp35974-scaffold431-2859919	48261592	12	48286143	48371815	7.93	TBC1D4
snp10013-scaffold1356-1211722	49467500	7	49495736	49510145	7.93	SPRY4
snp41806-scaffold543-4175450	106966316	3	107001107	107037825	7.92	DLGAP3
snp56583-scaffold894-1315674	13490477	30	13499025	13566622	7.92	TSC22D3
snp33904-scaffold4-769205	93111959	11	92985711	93476945	7.91	DENND1A
snp17755-scaffold1842-138629	3406415	11	3263373	3389534	7.90	TMEM131
snp41251-scaffold536-249647	7245974	11	7283269	7294454	7.88	MFSD9
snp41251-scaffold536-249647	7245974	11	7217329	7279675	7.88	SLC9A2
snp18784-scaffold19-4425409	64677932	30	64559560	64720979	7.88	BRWD3
snp55216-scaffold85-1273613	17381410	7	17392468	17396930	7.87	DIRAS1
snp55216-scaffold85-1273613	17381410	7	17357032	17364911	7.87	SGTA
snp55216-scaffold85-1273613	17381410	7	17377648	17384058	7.87	SLC39A3
snp55216-scaffold85-1273613	17381410	7	17333491	17345137	7.87	THOP1
snp5909-scaffold1206-678477	56393679	17	56382865	56414059	7.86	SUDS3
snp5909-scaffold1206-678477	56393679	17	56432040	56604592	7.86	TAOK3
snp8944-scaffold1322-658264	86012095	5	86038611	86053274	7.84	GOLT1B
snp8944-scaffold1322-658264	86012095	5	85956792	86014395	7.84	GYS2
snp8944-scaffold1322-658264	86012095	5	86053393	86089015	7.84	RECQL
snp8944-scaffold1322-658264	86012095	5	86020922	86029883	7.84	SPX
snp29140-scaffold3125-7445	116010625	3	115999109	116003080	7.81	CROCC2
snp36933-scaffold448-1433540	77550217	9	77583273	77792800	7.81	SCAF8
snp1354-scaffold1038-4862	844467	19	304128	1138942	7.80	CA10
snp41946-scaffold548-105829	26286316	15	26244237	26265309	7.79	BACE1
snp41946-scaffold548-105829	26286316	15	26276007	26345425	7.79	CEP164
snp41946-scaffold548-105829	26286316	15	26195625	26240724	7.79	RNF214
snp47339-scaffold665-1615171	34433186	5	34234029	34613705	7.77	NELL2
snp46783-scaffold653-529471	99520394	11	99500465	99535235	7.76	ABL1
snp46783-scaffold653-529471	99520394	11	99551823	99568400	7.76	FIBCD1
snp46783-scaffold653-529471	99520394	11	99540686	99541239	7.76	QRFP
snp24799-scaffold254-369527	57997743	7	57987431	57995580	7.74	CD74
snp24799-scaffold254-369527	57997743	7	58013484	58017420	7.74	RPS14
snp24799-scaffold254-369527	57997743	7	57947788	57985981	7.74	TCOF1

snp30658-scaffold339-4150649	45551987	7	45584757	45662792	7.74	FAM13B
snp30658-scaffold339-4150649	45551987	7	45523295	45542232	7.74	MYOT
snp30658-scaffold339-4150649	45551987	7	45543791	45586908	7.74	PKD2L2
snp17378-scaffold180-936947	18392023	3	18343701	18353604	7.72	ANXA9
snp17378-scaffold180-936947	18392023	3	18365079	18368950	7.72	CERS2
snp17378-scaffold180-936947	18392023	3	18333956	18343032	7.72	FAM63A
snp17378-scaffold180-936947	18392023	3	18368350	18398426	7.72	SETDB1
snp36349-scaffold4353-182963	116743421	3	116705004	116707709	7.70	PDCD1
snp36349-scaffold4353-182963	116743421	3	116724312	116728834	7.70	RTP5
snp43299-scaffold577-460316	14091055	5	14099258	14130076	7.70	TSPAN19
snp37183-scaffold452-1196129	19785356	19	19777250	19877833	7.70	MYO18A
snp37183-scaffold452-1196129	19785356	19	19748979	19762646	7.70	PIPOX
snp237-scaffold1006-645819	8405163	29	8374587	8401563	7.68	EED
snp18726-scaffold1895-259781	4523688	5	4560568	4601032	7.66	CAPS2
snp59320-scaffold975-1213737	12281623	23	12155806	12479531	7.65	DNAH8
snp10560-scaffold1376-1406813	73194248	8	73213947	73223629	7.65	BAG1
snp10560-scaffold1376-1406813	73194248	8	73226586	73240474	7.65	CHMP5
snp10560-scaffold1376-1406813	73194248	8	73198781	73206539	7.65	SPINK4
snp58279-scaffold945-1697505	108165663	1	108001492	108261942	7.64	VEPH1
snp20377-scaffold202-540677	30317420	2	30220911	30366138	7.63	SCN2A
snp54786-scaffold838-946181	12700195	28	12704559	12822609	7.63	PHYHIPL
snp52869-scaffold793-615567	47644946	11	47589844	47602264	7.61	CD8A
snp52869-scaffold793-615567	47644946	11	47612123	47652440	7.61	RMND5A
snp7708-scaffold1278-771909	68033839	13	68076423	68081782	7.60	EMILIN3
snp7708-scaffold1278-771909	68033839	13	68063762	68078064	7.60	LPIN3
snp7708-scaffold1278-771909	68033839	13	67927559	68059162	7.60	ZHX3
snp84-scaffold100-210601	5282247	15	5315124	5336553	7.59	BIRC2
snp84-scaffold100-210601	5282247	15	5206514	5284394	7.59	TMEM123
snp43306-scaffold577-708473	14339212	5	14375503	14397299	7.58	ALX1
snp43306-scaffold577-708473	14339212	5	14130161	14336237	7.58	LRRIQ1
snp6961-scaffold1256-213971	20422980	19	20461867	20567670	7.57	EFCAB5
snp6961-scaffold1256-213971	20422980	19	20220762	20399216	7.57	SSH2
snp22127-scaffold2185-306044	38500747	15	38470848	38534866	7.56	PARVA
snp14812-scaffold1599-477286	47745159	16	47721996	47801628	7.56	TTC34
snp29540-scaffold320-776697	147704741	1	147326883	147668469	7.55	KCNJ6
snp37288-scaffold454-1769470	40034626	20	39993599	40015234	7.51	TARS
snp43469-scaffold579-4675864	61143561	9	61147133	61373439	7.50	MAP3K5
snp43469-scaffold579-4675864	61143561	9	60960962	61117076	7.50	MAP7
snp9878-scaffold1352-1449195	92947590	6	92951090	92979876	7.50	BMP3
snp20458-scaffold202-4049754	33826497	2	33804660	33885573	7.50	DPP4
snp21197-scaffold207-3797821	16533442	17	16371646	16657845	7.49	RNF150
snp11591-scaffold1423-1776117	69396708	9	69419800	69482327	7.48	EPM2A
snp33728-scaffold397-62685	36047955	10	36083433	36084197	7.46	RPL10L
snp56039-scaffold873-1256692	21807282	30	21760010	21897069	7.44	DOCK11
snp13875-scaffold154-3493246	30519365	22	29982019	30513248	7.44	FOXP1
snp44176-scaffold60-601506	4936177	16	4915991	4942835	7.42	CFH
snp12376-scaffold1468-95374	23028157	21	23037082	23054290	7.41	CTSH
snp12376-scaffold1468-95374	23028157	21	23013066	23029887	7.41	MORF4L1
snp12376-scaffold1468-95374	23028157	21	23077136	23179266	7.41	RASGRF1
snp17499-scaffold181-371955	96270797	3	96237766	96252377	7.39	LRRC41
snp17499-scaffold181-371955	96270797	3	96306780	96323637	7.39	LURAP1
snp17499-scaffold181-371955	96270797	3	96252303	96278857	7.39	RAD54L
snp25952-scaffold2688-18597	66161285	21	66176048	66187150	7.37	ADSSL1
snp25952-scaffold2688-18597	66161285	21	66204164	66209442	7.37	AKT1
snp8941-scaffold1322-518308	85872139	5	85894650	85912973	7.37	LDHB
snp39736-scaffold509-45814	32727825	28	32741472	32748482	7.35	PPIF

snp39736-scaffold509-45814	32727825	28	32765340	32816779	7.35	ZCCHC24
snp39736-scaffold509-45814	32727825	28	32598941	32719158	7.35	ZMIZ1
snp32414-scaffold369-1265157	9005972	13	6976150	9059380	7.35	MACROD2
snp1728-scaffold1048-1443285	50775985	15	50665549	50726757	7.34	RNF169
snp1728-scaffold1048-1443285	50775985	15	50799183	50825381	7.34	SPCS2
snp1728-scaffold1048-1443285	50775985	15	50730616	50798706	7.34	XRRA1
snp10115-scaffold136-150821	23869363	29	23723499	23888528	7.31	NAV2
snp32202-scaffold3643-393290	49794943	19	49826987	49854929	7.31	ASPSCR1
snp32202-scaffold3643-393290	49794943	19	49748666	49755221	7.31	DUS1L
snp32202-scaffold3643-393290	49794943	19	49755414	49759668	7.31	GPS1
snp32202-scaffold3643-393290	49794943	19	49814286	49822101	7.31	LRRC45
snp32202-scaffold3643-393290	49794943	19	49811386	49813056	7.31	RAC3
snp32202-scaffold3643-393290	49794943	19	49760948	49764723	7.31	RFNG
snp32202-scaffold3643-393290	49794943	19	49824632	49825878	7.31	STRA13
snp54013-scaffold821-1145339	38828723	6	38387581	39698311	7.30	KCNIP4
snp41204-scaffold534-30258	51213263	18	51194626	51206364	7.29	IGSF23
snp41204-scaffold534-30258	51213263	18	51260402	51269445	7.29	NKPD1
snp41204-scaffold534-30258	51213263	18	51246606	51253443	7.29	PPP1R37
snp41204-scaffold534-30258	51213263	18	51222865	51238954	7.29	PVR
snp39993-scaffold510-1488579	5881940	7	5811604	5891583	7.28	NWD1
snp37032-scaffold449-759924	32993018	14	32948815	32957979	7.26	RP1
snp29908-scaffold325-568853	20979083	1	20877382	20977557	7.26	NRIP1
snp27536-scaffold294-2106	7129041	24	7093391	7121092	7.25	SOCS6
snp47875-scaffold675-232629	25466890	3	25515528	25588819	7.25	CASQ2
snp47875-scaffold675-232629	25466890	3	25451404	25451652	7.25	NHLH2
snp17406-scaffold1802-716394	26009025	5	25963434	25977216	7.23	AAAS
snp17406-scaffold1802-716394	26009025	5	25977486	25984534	7.23	C5H12orf10
snp17406-scaffold1802-716394	26009025	5	25989855	26011574	7.23	ESPL1
snp17406-scaffold1802-716394	26009025	5	26056222	26072841	7.23	ITGB7
snp17406-scaffold1802-716394	26009025	5	26017815	26019407	7.23	MFSD5
snp17406-scaffold1802-716394	26009025	5	25984898	25988485	7.23	PFDN5
snp17406-scaffold1802-716394	26009025	5	26035637	26055231	7.23	RARG
snp17406-scaffold1802-716394	26009025	5	25950262	25959197	7.23	SP7
snp40646-scaffold52-2617351	29574043	11	29622249	29653124	7.23	FBXO11
snp40646-scaffold52-2617351	29574043	11	29611692	29622279	7.23	MSH6
snp37948-scaffold47-1210340	55231175	30	55255117	55268894	7.22	DKC1
snp37948-scaffold47-1210340	55231175	30	55148092	55210480	7.22	GAB3
snp37948-scaffold47-1210340	55231175	30	55269980	55286799	7.22	MPP1
snp49011-scaffold7-2053765	30338229	13	30295240	30372727	7.22	TRDMT1
snp49011-scaffold7-2053765	30338229	13	30388146	30395383	7.22	VIM
snp29220-scaffold314-1763160	21764421	8	21705124	21734946	7.22	MTAP
snp47069-scaffold659-798157	21585507	3	21529785	21561514	7.21	PHGDH
snp25114-scaffold259-1662479	57922514	10	57824025	58099054	7.20	FBN1
snp6583-scaffold1234-345507	58119507	24	58140682	58149441	7.20	SEC11C
snp47462-scaffold6689-76365	100930047	5	100889134	100897084	7.18	C5H22orf23
snp47462-scaffold6689-76365	100930047	5	100865495	100886919	7.18	MICALL1
snp47462-scaffold6689-76365	100930047	5	100897149	100905880	7.18	POLR2F
snp47462-scaffold6689-76365	100930047	5	100909406	100913977	7.18	SOX10
snp59481-scaffold980-845143	83228568	6	83186636	83197701	7.17	AMBN
snp59481-scaffold980-845143	83228568	6	83222497	83240117	7.17	ENAM
snp59481-scaffold980-845143	83228568	6	83248737	83258709	7.17	JCHAIN
snp4737-scaffold1157-159806	113943596	3	113896009	113896589	7.14	HES6
snp4737-scaffold1157-159806	113943596	3	113904198	113932978	7.14	PER2
snp4737-scaffold1157-159806	113943596	3	113989839	114056619	7.14	TRAF3IP1
snp2923-scaffold109-662619	7312794	22	7362691	7399177	7.12	CRTAP
snp2923-scaffold109-662619	7312794	22	7245905	7321439	7.12	GLB1

snp2923-scaffold109-662619	7312794	22	7343703	7345307	7.12	TMPPE
snp7917-scaffold1287-141533	70243137	10	70157907	70308351	7.12	SYT16
snp58721-scaffold958-776977	21469623	13	21495707	21498809	7.12	SKIDA1
snp7711-scaffold1278-926214	67879534	13	67904050	67921918	7.11	PLCG1
snp7711-scaffold1278-926214	67879534	13	67820747	67874792	7.11	TOP1
snp20503-scaffold202-5963261	35740004	2	35709296	35815778	7.10	PLA2R1
snp45701-scaffold627-3512011	43213913	1	42861213	43258681	7.09	CMSS1
snp45701-scaffold627-3512011	43213913	1	42886833	43179948	7.09	FILIP1L
snp50511-scaffold725-1038375	66154228	9	66129081	66201172	7.09	VTA1
snp26010-scaffold27-205746	121107825	30	120715423	121123975	7.08	MID1
snp15875-scaffold1667-1154070	35491729	19	35449127	35455662	7.07	EME1
snp15875-scaffold1667-1154070	35491729	19	35436739	35448625	7.07	LRRC59
snp15875-scaffold1667-1154070	35491729	19	35455562	35460678	7.07	MRPL27
snp15875-scaffold1667-1154070	35491729	19	35466747	35474242	7.07	XYLT2
snp49862-scaffold712-214671	49928457	5	49788397	50353776	7.06	FAM19A2
snp41569-scaffold541-12945	53987778	6	53906188	53995063	7.06	DTHD1
snp10680-scaffold138-67757	105598322	2	105638787	105657922	7.05	ARPC2
snp10680-scaffold138-67757	105598322	2	105587091	105592060	7.05	CXCR1
snp10680-scaffold138-67757	105598322	2	105549665	105567474	7.05	CXCR2
snp32678-scaffold376-470308	51523875	16	51523484	51558642	7.05	PDPN
snp11125-scaffold14-2350075	69142718	14	69181015	69600910	7.04	ZFPM2
snp37644-scaffold463-676381	47242140	5	47180534	47268469	7.03	WIF1
snp29042-scaffold312-4303713	61727979	2	61685261	61748343	7.02	ACMSD
snp42272-scaffold556-600154	8608587	26	8526582	8628101	7.00	PTEN
snp52802-scaffold791-1809974	54459681	14	54374081	54579563	6.99	ZNF704
snp30797-scaffold340-42188	13492644	6	13372066	13453337	6.99	ENPEP
snp56794-scaffold9-1835501	60335356	4	60199278	60455060	6.99	BMPER
snp26075-scaffold271-591208	84522866	10	84560086	84563616	6.99	NGB
snp26075-scaffold271-591208	84522866	10	84572517	84605610	6.99	POMT2
snp26075-scaffold271-591208	84522866	10	84412260	84554245	6.99	TMEM63C
snp20517-scaffold202-6604247	36380990	2	36121178	36450399	6.97	BAZ2B
snp17183-scaffold1790-1268716	8240350	23	8266578	8272678	6.97	PACSIN1
snp17183-scaffold1790-1268716	8240350	23	8280496	8299503	6.97	SPDEF
snp29619-scaffold321-2228988	48255064	3	48198174	48206298	6.96	RPL5
snp51705-scaffold760-222846	7508063	6	7482866	7485010	6.96	TRAM1L1
snp12793-scaffold1490-44599	39752772	29	39756629	39795622	6.94	ATL3
snp12793-scaffold1490-44599	39752772	29	39669701	39703251	6.94	HRASLS5
snp12793-scaffold1490-44599	39752772	29	39697222	39706087	6.94	LGALS12
snp12793-scaffold1490-44599	39752772	29	39795757	39866012	6.94	RTN3
snp56719-scaffold898-253921	5151423	4	5122812	5509351	6.94	ABCA13
snp51110-scaffold746-55079	41436884	17	41463139	41557739	6.93	GRIA2
snp55911-scaffold869-1796393	31694359	16	31591143	31726869	6.92	CEP170
snp10028-scaffold1356-1850057	48829165	7	48770514	48886731	6.92	DIAPH1
snp44739-scaffold609-408841	50019100	17	49842867	50239942	6.92	TMEM132B
snp20653-scaffold2034-689378	689378	29	595533	639749	6.91	CEP295
snp34270-scaffold402-1953496	110391497	30	110431222	110655298	6.90	CNKSR2
snp34270-scaffold402-1953496	110391497	30	110428773	110433236	6.90	KLHL34
snp34270-scaffold402-1953496	110391497	30	110317182	110367234	6.90	SMPX
snp40818-scaffold524-844497	25887374	9	25849366	25904409	6.89	FYN
snp59468-scaffold980-522581	82906006	6	82894746	82908392	6.89	CSN3
snp5067-scaffold1172-113725	35491803	28	34431805	35658169	6.89	NRG3
snp59167-scaffold971-125501	142272452	1	142301577	142319224	6.88	UBE2G2
snp26751-scaffold281-747096	35081977	6	35081521	35095015	6.87	IBSP
snp26751-scaffold281-747096	35081977	6	35045422	35059786	6.87	MEPE
snp59100-scaffold969-3176120	29753867	9	29684804	29956565	6.87	PDSS2
snp3355-scaffold1102-483452	38094943	28	37888788	38577268	6.87	GRID1

snp55536-scaffold860-695794	17575017	4	17360213	17848796	6.87	THSD7A
snp53867-scaffold82-7642345	78844910	2	78759491	78832020	6.86	GLS
snp53867-scaffold82-7642345	78844910	2	78834675	78877613	6.86	STAT1
snp53867-scaffold82-7642345	78844910	2	78885554	78989435	6.86	STAT4
snp29470-scaffold3193-172583	54759606	18	54800146	54810633	6.86	IZUMO2
snp29470-scaffold3193-172583	54759606	18	54719224	54756347	6.86	VRK3
snp29470-scaffold3193-172583	54759606	18	54756801	54768765	6.86	ZNF473
snp11979-scaffold1440-432774	108825731	6	108761413	109229113	6.86	LDB2
snp58103-scaffold94-5833878	68731943	6	68682418	68729268	6.86	KDR
snp2010-scaffold1059-936497	130673358	2	130571058	130636651	6.84	ECE1
