# Supplementary material for: Selection Signatures Analysis Reveals Genes Associated with High-Altitude Adaptation in Tibetan Goats from Nagqu, Tibet
Source: Animals (Basel). 2020 Sep 8;10(9):1599. doi: 10.3390/ani10091599 (PMC7552128; doi:10.3390/ani10091599)
Supplement: Supplementary file 1 [file animals-10-01599-s001.zip › Supplement Table/Table S2.pdf]

Gene	Prime(5'to3')
LEPR-F	GGAGCGCCCTTCTTACCTTT
LEPR-R	GTTAGACCCAACCGCTGTCA
LDB1-F	TTATGTCCCGCCACAAGACC
LDB1-R	CCACCACCATCACATCCTGG
EGFR-F	GCAGAACGAAGCAACATGGC
EGFR-R	GATGAGCACAGTGGGTGACA
FGF2-F	GGCCACTTTAAGGACCCCA
FGF2-R	GAAGTTGTAGTTTGATGTGAGGG
